# 1687. Antimicrobial Activity of Ceftibuten-Avibactam against a Global Collection of Enterobacterales from Patients with Complicated Urinary Tract Infections (2021)

**DOI:** 10.1093/ofid/ofac492.1317

**Published:** 2022-12-15

**Authors:** Helio S Sader, Cecilia G Carvalhaes, Michael D Huband, Rodrigo E Mendes, Mariana Castanheira

**Affiliations:** JMI Laboratories, North Liberty, Iowa; JMI Laboratories, North Liberty, Iowa; JMI Laboratories, North Liberty, Iowa; JMI Laboratories, North Liberty, Iowa; JMI Laboratories, North Liberty, Iowa

## Abstract

**Background:**

Limited therapeutic options are currently available for oral treatment of complicated urinary tract infections (cUTIs) caused by resistant Enterobacterales (ENT). Ceftibuten (CTB) is an oral cephalosporin active against ENT approved by the US FDA in 1995. Avibactam (AVI) is a potent inhibitor of extended-spectrum β-lactamases (ESBLs), serine carbapenemases, and AmpC that can be administered orally. We evaluated the *in vitro* activity of CTB-AVI against ENT causing cUTI.

**Figure:**
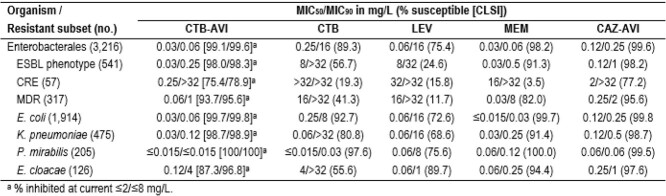

**Methods:**

3,216 isolates (1/patient) were consecutively collected from patients with cUTI in 72 hospitals from 25 countries in 2021 then susceptibility (S) tested by CLSI broth microdilution. Isolates were mainly from the US (*n*=1,584; 29 centers) and Europe (*n*=1,411; 33 centers in 18 countries), but included *E. coli* isolates from Latin America (LATAM; *n*=121; 6 centers in 5 countries) and Japan (*n*=100). A proposed CTB-AVI breakpoint (≤ 2 mg/L) and the current CLSI breakpoint for CTB (≤ 8 mg/L) were applied for comparison.

**Results:**

The most active agents against ENT were CTB-AVI (99.1%/99.6% inhibited at ≤ 2/≤ 8 mg/L), ceftazidime (CAZ)-AVI (99.6%S), amikacin (AMK; 99.1%S), and meropenem (MEM; 98.2%S; Table). CTB-AVI was 4-fold more potent than CAZ-AVI based on MIC_50/90_ values. The most active oral agents were CTB (89.3%S; 82.9% inhibited at ≤ 2 mg/L), levofloxacin (LEV; 75.4%S), and trimethoprim-sulfamethoxazole (TMP-SMX; 73.4%S). Only CTB-AVI, CAZ-AVI, MEM, and AMK were active against >90% of ESBL-phenotype isolates; only CTB-AVI, CAZ-AVI, and AMK were active against > 70% of CRE isolates. CRE rates were 1.8%/1.9% in US/EU; CTB-AVI inhibited 79.3%/81.5% of CRE isolates from US/EU at ≤ 8 mg/L (75.9%/77.8% at ≤ 2 mg/L). The second most active oral agent against CRE was TMP-SMX (24.6%S). Only 17 isolates (0.5%) showed CTB-AVI MICs ≥8 mg/L; these were from the US (*n*=9; 0.6% of US isolates), EU (*n*=6; 0.4%), and LATAM (*n*=2; 1.7%). Among these 17 isolates, 23.5% were MEM-S, 41.2% were TMP-SMX-S, and 82.4% were AMK-S.

**Conclusion:**

CTB-AVI was highly active against a large collection of contemporary ENT isolated from patients with cUTIs and exhibited a similar spectrum to CAZ-AVI. CTB-AVI may represent a valuable option for oral treatment of cUTI caused by resistant ENT.

**Disclosures:**

**Helio S. Sader, MD, PhD**, AbbVie: Grant/Research Support|Cidara: Grant/Research Support|Melinta: Grant/Research Support|Nabriva Therapeutics: Grant/Research Support|Pfizer: Grant/Research Support **Cecilia G. Carvalhaes, MD, PhD**, AbbVie: Grant/Research Support|Cidara: Grant/Research Support|Melinta: Grant/Research Support|Pfizer: Grant/Research Support **Michael D. Huband, BS**, Pfizer: Grant/Research Support **Rodrigo E. Mendes, PhD**, AbbVie: Grant/Research Support|Cidara: Grant/Research Support|GSK: Grant/Research Support|Melinta: Grant/Research Support|Nabriva Therapeutics: Grant/Research Support|Office for Assistant Secretary of Defense for Health Affairs: Grant/Research Support|Pfizer: Grant/Research Support|Shionogi: Grant/Research Support|Spero Therapeutics: Grant/Research Support **Mariana Castanheira, PhD**, AbbVie: Grant/Research Support|Cidara: Grant/Research Support|GSK: Grant/Research Support|Melinta: Grant/Research Support|Pfizer: Grant/Research Support|Shionogi: Grant/Research Support.

